# A scoping review of mystical-type experiences and mood symptom outcomes in psychedelic therapy clinical trials: comparing life-threatening disease and depressive populations

**DOI:** 10.1177/20451253261433836

**Published:** 2026-03-27

**Authors:** Ana Deutsch, Luis E. Contreras, Sarah Kratina, Leah M. Mayo

**Affiliations:** Mathison Centre for Mental Health Research and Education, University of Calgary, Calgary, AB, Canada; Hotchkiss Brain Institute, University of Calgary, Calgary, AB, Canada; Department of Psychiatry, Cumming School of Medicine, University of Calgary, Calgary, AB, Canada; Mathison Centre for Mental Health Research and Education, University of Calgary, Calgary, AB, Canada; Hotchkiss Brain Institute, University of Calgary, Calgary, AB, Canada; Department of Psychiatry, Cumming School of Medicine, University of Calgary, Calgary, AB, Canada; Mathison Centre for Mental Health Research and Education, University of Calgary, Calgary, AB, Canada; Hotchkiss Brain Institute, University of Calgary, Calgary, AB, Canada; Department of Psychiatry, Cumming School of Medicine, University of Calgary, Calgary, AB, Canada; Mathison Centre for Mental Health Research and Education, University of Calgary, Calgary, AB, Canada; Hotchkiss Brain Institute, University of Calgary, Calgary, AB, Canada; Department of Psychiatry, Cumming School of Medicine, University of Calgary, 2500 University Drive, Calgary, AB T2N 1N4, Canada

**Keywords:** anxiety, depression, life-threatening disease, mystical experiences, psychedelics

## Abstract

**Background:**

Psychedelic therapies are gaining attention as tools to alleviate anxiety and depression across various clinical populations. However, the mechanisms behind psychedelics’ therapeutic efficacy and the potential differences in how patients with certain diagnoses experience their subjective effects remain unknown. One commonly suggested mediator of positive outcomes across psychedelics trials is the occurrence of mystical-type experiences.

**Objectives:**

This scoping review examines the relationship between psychedelic-induced mystical-type experiences and changes in anxiety and depression symptoms, comparing findings across populations with a life-threatening disease (LTD) and other psychiatric populations. Given the unique challenges faced by patients with an LTD diagnosis, this review aimed to determine whether there are any distinct patterns differentiating the effects of mystical-type experiences and mood outcomes in this population from other psychiatric populations.

**Charting methods:**

Following a scoping review method, PubMed, Embase, and PsycINFO were reviewed.

**Eligibility criteria:**

Clinical trials administering psychedelics to adults and measuring mystical-type experiences and their relationship to anxiety and/or depression outcomes were included.

**Sources of evidence:**

Thirteen clinical trials (*n* = 410 participants) met the inclusion criteria. Five studies administered psychedelic therapy to LTD populations, eight trials administered psychedelic therapy to patients with depression.

**Results:**

Across all studies, 69% of trials reported a positive relationship between mystical-type experiences and improvement in anxiety and/or depression outcomes. This relationship was found in 80% of LTD studies and 63% of studies in depressive populations.

**Conclusion:**

Mystical-type experiences were commonly associated with reductions in anxiety and/or depression symptoms following psychedelic therapy in both LTD and depressive populations. However, this relationship may depend on multiple factors, including the timing of symptom assessments and therapeutic context. Future studies should examine the variables that affect mystical-type experiences, along with other aspects of set and setting, to determine how to best facilitate positive outcomes induced by psychedelics.

## Introduction

Psychedelic drugs are increasingly being studied for the treatment of various psychiatric disorders, including major depressive disorder (MDD),^1–3^ substance use disorders,^4,5^ and end-of-life anxiety and depression.^6,7^ While various theories highlight the importance of different aspects of the psychedelic experience in driving clinical outcomes, the exact mechanisms behind their therapeutic effects remain largely unknown. Some believe that the subjective effects of psychedelics are an important and even necessary factor in facilitating positive outcomes,^8^ although this remains a topic of debate in the field.^9^

This debate becomes more complicated when we consider the differences in how psychedelics are defined and categorized. Generally, psychedelics are a class of drugs that produce alterations in perception, mood, and cognition.^10^ “Classical,” or serotonergic, psychedelics have a common mechanism of action and are agonists or partial agonists of serotonin 2A receptors (5-HT_2A_-R).^10^ This classification includes drugs such as psilocybin, lysergic acid diethylamide (LSD), mescaline, and *N,N*-dimethyltryptamine (DMT). However, other compounds with different pharmacology that produce profound alterations in perception are often also categorized as psychedelics. This includes dissociative anesthetics such as ketamine, stimulant-empathogen drugs such as 3,4-methylenedioxymethamphetamine (MDMA), and atypical psychedelics like ibogaine. In this review, we focus on the subjective effects associated with a broad range of substances commonly referred to as psychedelics, including both classical and non-classical psychedelics.

Among the various subjective effects associated with psychedelics, one that has gained significant attention in clinical trials is the mystical-type experience. Common conceptualizations of mystical-type experiences are largely based on philosopher Walter Stace’s work, who claimed that all mystical experiences have certain common features, including a sense of unity, sacredness, noetic quality, deeply felt positive mood, ineffability, paradoxicality, and transcendence of time and space.^11^ In clinical trials investigating psychedelics, researchers use questionnaires to quantify these aspects of mystical-type experiences. The Mystical Experiences Questionnaire (MEQ) is the most frequently used tool in modern psychedelic research, originally developed by Griffiths and colleagues.^12^ While the original 43-item MEQ is scored into seven factors corresponding to Stace’s dimensions of mystical experiences, the revised 30-item MEQ (MEQ-30) condenses these into four core factors: mystical aspects (including internal unity, external unity, noetic quality, and sacredness), positive mood, transcendence of time and space, and ineffability.^13^ The MEQ-30 has been validated using data from controlled studies administering psilocybin. Other tools used to assess mystical-type experiences include the oceanic boundlessness subscale of the 5-Dimensional Altered States of Consciousness questionnaire (5D-ASC) and the Ego Dissolution Inventory (EDI).^14^

These measures are especially relevant in psychedelic-assisted therapy, where mystical-type experiences are proposed to be associated with improvements in clinical outcomes. For example, in patients with tobacco use disorder, scores on the MEQ correlated with abstinence and reductions in cravings.^15^ Similarly, in patients with MDD, scores on the MEQ had a significant and moderate correlation to decreases in depression scores.^2^ One area where these findings have garnered particular attention is in the treatment of depression and anxiety in patients facing life-threatening diagnoses such as cancer.^6,7^ Depression and anxiety disorders are common in this patient population, and are linked with poor outcomes, including reduced quality of life, lower treatment adherence, and decreased survival rates.^16,17^ Historical studies on the use of LSD to alleviate emotional distress in cancer patients^18^ may have influenced the resurgence of modern psychedelic-assisted therapy in populations with a life-threatening disease (LTD). Notably, several modern trials have found that mystical-type experiences induced by serotonergic psychedelics, including psilocybin and LSD, play a mediating role in reducing depression and anxiety in this population.^6,7,19^

Previous reviews have investigated the relationship between mystical-type experiences induced by psychedelics and various outcomes–for example, improvements in well-being and mental health,^20^ and symptom reduction in various mental health disorders.^21^ While the literature presents some mixed findings, many studies found a positive relationship between mystical-type experiences and increases in well-being and mental health.^20^ However, most studies reporting this association were conducted in healthy populations, raising the question of whether mystical-type experiences are necessary for observing improvements in symptoms in clinical populations. Although a review conducted in clinical populations found that the majority of studies report a positive correlation between mystical-type experiences and symptom improvement, half of the included studies were open-label, creating a high potential for biased results.^21^ The inclusion of more randomized, placebo-controlled trials, in addition to open-label trials, is imperative for drawing conclusions about the importance of these experiences in clinical populations. Moreover, the existing literature lacks a comprehensive review aimed at understanding the differences in how mystical-type experiences might impact treatment outcomes in specific clinical populations.

Although mystical-type experiences might serve as a common mediator of positive treatment outcomes across clinical populations, it is imperative to investigate whether specific features of these experiences are more relevant to certain diagnoses. At present, whether mystical-type experiences function similarly across different populations, or whether they are more strongly linked to symptom improvements in specific patient cohorts, is unknown. LTD patients facing depression and anxiety may process these experiences differently from individuals diagnosed with depression or anxiety disorders, given the unique psychological and existential challenges associated with terminal illness.^22^ In cancer patients, the occurrence of psychedelic-induced mystical-type experiences has been shown to facilitate a reconciliation between illness, mortality, and the meaning of life, as well as to promote a softening of rigid psychological patterns and the burden of their diagnosis.^23^ Importantly, the study reporting this^23^ did not formally measure mystical-type experiences, but qualitatively analyzed participants’ narratives from a psilocybin RCT reported by Ross et al.^7^ In this context, psychedelic therapy may lead to unique experiences in LTD populations by allowing patients to face their diagnosis and possibly find new meaning in their lives, enabling an acceptance of mortality without overwhelming fear, anxiety, and depression. On the other hand, mystical-type experiences may have different features and implications in people who are not faced with a terminal diagnosis, but who suffer from depression and anxiety. By exploring how psychedelic-induced mystical-type experiences are associated with therapeutic outcomes in individuals experiencing distress due to a life-threatening disease and in other clinical populations with anxiety and depression, this review maps the characteristics and reported mood effects of these experiences across different populations. Identifying potential differences in how mystical-type experiences relate to relief from anxiety and depression may help inform future hypotheses about the mechanisms through which psychedelic therapies exert their therapeutic effects, and whether different clinical approaches may be warranted depending on the nature of the anxiety and/or depression.

This scoping review focuses on the relationship between mystical-type experiences induced by psychedelics as they relate to anxiety and depression outcomes, comparing LTD populations with other clinical populations. Given the variability seen in naturalistic and observational studies, we have chosen to limit this review specifically to clinical studies administering controlled doses of psychedelics and measuring the occurrence of mystical-type experiences.

## Methods

**Figure 1.**
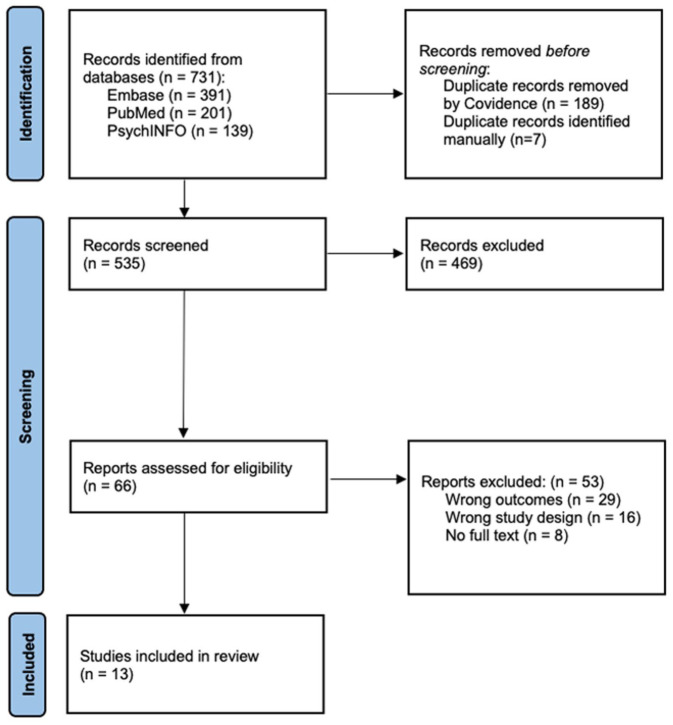
Preferred reporting items for systematic reviews and meta-analysis (extension modified for scoping reviews) diagram of the search strategy.

The scoping review followed an established framework^24^ with updated methodological guidance.^25^ The completed Preferred Reporting Items for Systematic Review and Meta-Analyses extension for scoping reviews (PRISMA-ScR) guideline (Supplemental Material)^26^ tracked the reporting of key elements of the review process (Figure 1).

### Stage 1: Identifying the research question

The research question guiding this review is: What is the relationship between mystical-type experiences and anxiety and depression treatment outcomes in life-threatening disease populations compared with other clinical populations?

### Stage 2: Identifying relevant studies

APA PsycINFO, Embase, and PubMed were searched from any publication date to July 24, 2024. The three search concepts included were: mystical-type experiences, psychedelics, and mood symptoms. The search was conducted in all fields and multipurpose, with “AND” used to link the three concepts.

### Stage 3: Study selection

The included studies recruited adult participants (age ⩾ 18 years) who experienced symptoms of anxiety and/or depression or were clinically diagnosed with anxiety and/or depression. Interventions administered were those that used any type of psychedelics, including psilocybin, LSD, ayahuasca, DMT, MDMA, mescaline, and ibogaine within a clinical context (e.g., research center and hospital). Only full-text clinical trials published in English in peer-reviewed journals were included. Gray literature, conference abstracts, poster presentations, and dissertations were excluded. Naturalistic studies were excluded, as were those including a healthy population.

Results from each database search were exported and uploaded into Covidence for data management and data screening. Duplicates were removed through Covidence. Three members of the research team independently screened the titles and abstracts of all the imported studies. Articles that fulfilled the study selection criteria were included in the full-text review phase. The search strategy produced 731 records, and 13 clinical trials were included in the scoping review (see Figure 1).

### Stage 4: Charting the data

Data were extracted by three members of the study team—Member 1, Member 2, and Member 3 using a data extraction form developed by Member 1 and revised by Member 2 and Member 3. The data extraction form included the following key variables: author, year of publication, study design, type of psychedelic, participants’ mental health clinical indication(s), mood symptom measures, mystical-type experience measures, and type of relationship between mood symptom(s) and mystical-type experiences. Discrepancies were resolved through discussion with Members 1–3.

### Stage 5: Collating, summarizing, and reporting the results

Data related to study characteristics, population details, intervention details, and outcomes were extracted (see Table 1). Results were summarized using quantitative descriptors and further interpreted in relation to existing literature in the discussion.

**Table 1.** Synthesis of included studies.

Paper	Study design	Clinical population	Sample size	Type of psychedelic	Dose	Therapy (Y/N)	Mood symptom measures	Mystical-type experience measures	Outcome
End-of-life populations									
Ross et al., 2016	Randomized controlled trial	Cancer-related anxiety diagnosis and depression	29	Psilocybin	0.3 mg/kg (oral)	Y	HADS-A, STAI, HADS-D, BDI	MEQ30	Reductions in anxiety (measured by STAI and HADS-A) and depression symptoms (measured by HADS-D and BDI) correlated with higher mystical-type experience scores (measured by MEQ)
Agin-Liebes et al., 2020 (follow-up to Ross et al., 2016)	Long-term within-subject follow-up observational design	Cancer-related symptoms or diagnosis of anxiety and depression	14/29 above participants	Psilocybin	N/A		HADS, STAI, BDI	MEQ-30	No relationship between changes in depression (measured by BDI, HADS) or anxiety (measured by STAI, HADS) and mystical-type experience scores (measured by MEQ)
Griffiths et al., 2016	Randomized controlled trial	Cancer patients with symptoms or diagnosis of depression and/or anxiety	56	Psilocybin	22 or 30 mg/70 kg (oral)—two sessions, counterbalanced	Y	HADS, HAM-A, STAI, GRID-HAMD-17, BDI	5D-ASC, Mysticism Scale (nine-points), HRS, MEQ30	Decreases in depression (measured by GRID-HAMD, BDI, HADS-D) and anxiety (measured by HAM-A, STAI-Trait, HADS-A) correlated with higher mystical-type experience scores (measured by MEQ)
Holze et al., 2023	Randomized controlled trial	Anxiety symptoms with or without a life-threatening somatic illness	42 (20 LTD patients)	LSD	2X 200 µg (oral)	Y	STAI-Global, HAM-D, BDI	5D-ASC, MEQ30	Decreases in anxiety symptoms (measured by STAI-G) correlated with higher mystical-type experience scores (measured by MEQ) and higher oceanic boundlessness (measured by 5D-AC)
Lewis et al., 2023	Open-label clinical trial	Cancer patients with a depressive disorder	12	Psilocybin	25 mg (oral)	Y	HAM-D	MEQ30	Decreases in depressive symptoms (measured by HAM-D) were correlated with higher mystical-type experience scores (measured by MEQ-Total, MEQ-Mystical, and MEQ-Ineffability)
Other clinical populations									
Sumner et al., 2021	Randomized controlled trial	Moderate to severe major depressive disorder	32	Ketamine	0.44 mg/kg (intravenous)	N	MADRS	11D-ASC	Decreases in depressive symptoms (measured by MADRS) were correlated with higher mystical-type experience scores (measured by the unity, spirituality, and insight factors of the 11D-ASC)
Gukasyan et al., 2022	Randomized, waiting-list controlled trial	Unipolar depression	27	Psilocybin	20 mg/70 kg and 30 mg/70 kg (oral)	Y	HAM-D, BDI	MEQ30	No relationship between depressive symptoms (measured by HAM-D & BDI) and mystical-type experiences (measured by MEQ30)
Levin et al., 2024	Randomized, waiting-list controlled trial	Moderate to severe major depressive disorder	24	Psilocybin	20 mg/70 kg and 30 mg/70 kg (oral)	Y	GRID-HAMD	MEQ30	Reductions in depressive symptoms (measured by HAM-D) correlated with higher mystical-type experience scores (measured by MEQ30)
Murphy et al., 2022	Randomized controlled trial	Moderate to severe major depressive disorder	59	Psilocybin	25 mg 2x (oral)	Y	QIDS	MEQ30	Reductions in depressive symptoms (measured by QIDS) correlated with higher mystical-type experience scores (measured by MEQ30)
Palhano-Fontes et al., 2019	Randomized controlled trial	Treatment-resistant major depressive disorder	29	Ayahuasca	(mean ± s.d.) 0.36 ± 0.01 mg/ml of *N,N*-DMT, 1.86 ± 0.11 mg/ml of harmine, 0.24 ± 0.03 mg/ml of harmaline, and 1.20 ± 0.05 mg/ml of tetrahydroharmine (oral)	N	HAM-D, MADRS	MEQ30	Worsening depressive symptoms (measured by MADRS) correlated to higher scores on the subscale of transcendence of time and space (measured by MEQ30). Other mystical experience factors were not corelated with mood.
Roseman et al., 2018	Open-label clinical trial	Treatment-resistant, moderate to severe major depression	20	Psilocybin	10 mg or 25 mg (oral)	Y	STAI, QIDS, BDI, HAM-D	5D-ASC	Reduction in depressive symptoms (measured by QIDS) correlated with higher mystical-type experience scores (measured by OBN subscale of 5D-ASC)
Sloshower et al., 2023	Exploratory placebo-controlled, within-subject, fixed-order study	Major depressive disorder	19	Psilocybin	0.3 mg/kg (oral)	Y	HAM-A, GRID-HAM-D, QIDS	MEQ30	Reductions in depressive symptoms (measured by HAM-D) were correlated with higher mystical-type experience scores (measured by MEQ30) in the placebo session, but not in the psilocybin session
Weiss et al., 2024	Randomized controlled trial	Major depressive disorder	59	Psilocybin		N/A	BDI-IA, HAM-D, MADRS, QIDS	MEQ, EDI	Reductions in depressive symptoms (measured by HAM-D) correlated with higher mystical-type experience scores (MEQ) and greater EDI

11D-ASC, 11-Dimensional Altered States of Consciousness Scale; 5D-ASC, 5-Dimensional Altered States of Consciousness Scale; BDI, Beck’s Depression Inventory; EDI, Ego dissolution inventory; GRID-HAMD-17, GRID Hamilton Depression Rating Scale; HADS, Hospital Anxiety and Depression Scale; HADS-A, HADS – Anxiety; HADS-D, HADS – Depression; HAM-A, Hamilton Anxiety Rating Scale; HAM-D, Hamilton Depression Rating Scale; HRS, Hallucinogen Rating Scale; MADRS, Montgomery–Åsberg Depression Rating Scale; MEQ30, 30-item Mystical Effects Questionnaire; QIDS, Quick Inventory of Depressive Symptomatology; STAI, State and Trait Anxiety Inventory.

## Results

There were 13 clinical trial studies published between 2016 and 2024 included in this scoping review. Table 1 provides a summary of all the included studies.

### Study characteristics

#### Study design

Sixty-nine percent (*n* = 9/13) of these studies were randomized controlled trials (RCTs); two (22%; *n* = 2/9) of these RCTs were randomized waitlist trials. Of note, one of the RCTs^27^ was a long-term follow-up to another trial that was included in this review.^7^

### Population details

#### Study populations

The results identified two study populations:

(1) patients diagnosed with depression and/or anxiety associated with an LTD

In LTD populations, five of the total studies included (38%; *n* = 5/13) examined anxiety and depression symptoms in participants with either a cancer diagnosis or another life-threatening illness.

(2) patients diagnosed with a depressive disorder that measured the relationship between anxiety and/or depression symptoms and mystical-type experiences.

The remaining eight of the total studies (62%; *n* = 8/13) included non-LTD populations: of those, 31% (*n* = 4/13) of studies included participants with MDD^28–31^; two (15%) studies included participants with treatment-resistant depression^32,33^; two (15%) studies included participants with symptoms of moderate to severe depression.^34,35^

#### Sample size

*LTD:* In the first study population, the clinical trials included *N* = 141 participants with an LTD diagnosis with anxiety and/or depressive symptoms.
*Depressive:* In the second study population, the trials included *N* = 269 participants who had a depressive disorder not associated with an LTD diagnosis.

Overall, across the two trial categories, the number of participants ranged from 12 participants to 59 participants, with a total of *N* = 410 across all the trials.

### Intervention details

#### Type of psychedelic substance

Across all trial populations the majority of included studies (*n* = 10/13) administered psilocybin, one study administered ayahuasca,^32^ one study administered LSD,^19^ and one study administered ketamine.^28^

*LTD:* Of the five studies conducted in LTD populations, the majority (*n* = 4/5) administered psilocybin, and one administered LSD.^19^
*Depressive:* Of the eight studies conducted in depressive populations, the majority (*n* = 6/8) administered psilocybin, one administered ayahuasca,^32^ and one administered ketamine.^28^

#### Psychotherapeutic support

*LTD:* All studies in LTD populations included psychotherapeutic support.
*Depressive:* The majority of studies in depressive populations without an LTD diagnosis included psychotherapeutic support (75%, *n* = 6/8), while two studies did not.^28,32^

Overall, psychotherapeutic support was included in the majority of studies reviewed (85%; *n* = 11/13).

#### Dosing setting

*LTD:* Of the studies in LTD populations, one provided psilocybin-assisted group psychotherapy, administering the psilocybin within an infusion suite that had private bays, with a private breakout room available.^36^
*Depressive:* Of the studies in depressive patients, one administered ketamine infusions in an MRI environment.^28^

Aside from these two exceptions, the studies generally administered the psychedelic dose in home-like aesthetic environments with artwork, plants, soft lighting, and personal items. One study was a non-interventional follow-up to a clinical trial, and thus did not refer to the dosing setting,^27^ but this was described in the original trial.^7^

### Outcomes

#### Anxiety and depression outcome measures

*LTD:* All but one^36^ of the studies in LTD populations (*n* = 4/5) measured anxiety symptoms using the state and trait anxiety inventory (STAI). Depression symptoms were assessed in all LTD studies. The most commonly used scale to assess depression was the Beck’s Depression Inventory (BDI), administered in most of the LTD studies^6,7,19,27^ (*n* = 4/5). The Hamilton Depression Rating (HAM-D) scale was administered in two studies^19,36^ and the Hospital Anxiety and Depression Scale (HADS) was administered in two studies.^7,27^
*Depressive:* Of the studies in depressive populations, two measured anxiety, and used the STAI^33^ and the Hamilton Anxiety Rating Scale (HAM-A).^30^ All studies employed some measure of depressive symptoms, with the HAM-D and the Quick Inventory of Depressive Symptomatology (QIDS) being the most common. The HAM-D was administered in half of the studies (*n* = 4/8)^29,32,33,36^ and the QIDS was also administered in half of the studies (*n* = 4/8).^29,30,33,34^ Other commonly used scales were the BDI (*n* = 3/8)^29,33,35^ and the Montgomery–Åsberg Depression Rating Scale (MADRS), which was administered in 3/8 studies in this population.^28,29,32^

Overall, about half (*n* = 6/13) of all studies measured anxiety symptoms, and all (100%; *n* = 13/13) studies measured depressive symptoms. The BDI was the most frequently used measure across all studies (54%; *n* = 7/13).

#### Mystical-type experience outcomes measures

Across all trial populations, the most frequently used mystical-type experience measure was the MEQ-30 (85%; *n* = 11/13), followed by the 5D-ASC (23%; *n* = 3/13). Other mystical-type experience measures included the 11D-ASC, the Mystical Scale (9-points), the Hallucinogen Rating Scale (HRS), and the EDI. Although the included studies used different validated instruments to assess mystical-type experiences, these tools are generally considered to capture facets of the same higher-order construct. For this reason, we synthesized them under the broader category of “mystical-type experiences,” while specifying in Table 1 which measure was used in each study.

#### Relationship between clinical symptoms and mystical-type experiences

*LTD:* Of the five studies in LTD populations, four (80%) identified a positive relationship between mystical-type experiences and mood symptoms, and one (20%) study found no relationship.^27^ Of note, the study that found no significant relationship was a long-term follow-up to another one of the included studies.^7^
*Depressive:* Of the eight studies in depressive populations, five (63%) found a positive relationship, one (13%) found a negative relationship,^32^ one (13%) displayed mixed findings,^30^ and one study (13%) found no significant relationship.^35^ In the study that found a negative relationship, increases in depression symptoms were correlated with the transcendence of time and space subscale of the MEQ.^32^ Other factors of mystical-type experiences were not corelated with changes in depression. In the study displaying mixed findings, MEQ scores were not significantly correlated with improvements in depression scores after the psilocybin sessions, but MEQ scores were significantly correlated with changes in depression scores at both 1 day and 2 weeks after the placebo sessions.^30^

Overall, the majority (69%; *n* = 9/13) of studies in both populations identified a positive relationship between the magnitude of mystical-type experiences and improvement in anxiety and/or depression outcomes.

#### Subscales of mystical-type experiences

*LTD:* Of the studies in LTD patients, one study assessed the contribution of specific dimensions of mystical experiences to treatment outcomes and found that the ineffability and mystical subscales of the MEQ were positively correlated with changes in depression scores.^36^ The same study found that participants who had a “complete” mystical experience had greater decreases in depression than those who did not.
*Depressive:* Of the studies in depressive populations, one found a negative correlation between the transcendence of time and space subscale of the MEQ and changes in depression.^32^ One other study found that the 11D-ASC dimensions of spirituality, experience of unity, and insight were correlated with the magnitude of change in depression scores.^28^

#### Assessment time point

Psychedelic-induced mystical-type experiences were assessed at the end of the dosing day in all of the studies. However, clinical mood symptoms were assessed at different time points across the studies reviewed, as can be seen in Table 2.

**Table 2.** Summary of when the relationship between mystical experiences and treatment outcomes was assessed across the studies reviewed.

Study	Time point (relative to dosing)	Relationship (Y/N)
End-of-life populations		
Agin-Liebes et al., 2020	4.5 Years (second long-term follow-up)	N
Griffiths et al., 2016	5 Weeks	Y
Holze et al., 2023	16 Weeks	Y
Lewis et al., 2023	2 Weeks	Y
Ross et al., 2016	6 Weeks	Y
Other clinical populations		
Sumner et al., 2021	24 h	Y
Gukasyan et al., 2022	4 Weeks	N
	3 Months	N
	6 Months	N
	12 Months	N
Levin et al., 2024	4 Weeks	Y
	3 Months	N
	6 Months	N
	12 Months	N
Murphy et al., 2022	6 Weeks	Y
Palhano-Fontes et al., 2019	7 Days	Y
Roseman et al, 2018	1 Day	Y
	1 Week	Y
	5 Weeks	Y
	3 Months	Y
	6 Months	Y
Sloshower et al., 2023	1 Day	N—after psilocybin. Y—after placebo
	1 Week	N
	2 Weeks	N—after psilocybin. Y—after placebo
Weiss et al., 2024	6 Weeks	Y

The relationship column refers to whether or not the study found a significant relationship between mystical experiences and changes in depression/anxiety at the specific time points when the relationship was assessed.

*LTD:* The time of assessment ranged from 2 weeks^36^ to 4.5 years,^27^ with all studies employing a different time point. One study employed multiple time points and found that the relationship that was significant at the first assessment^7^ was no longer significant at the follow-up 4.5 years later.^27^
*Depressive:* The time of assessment ranged from 24 h^28^ to 12 months.^31,35^ Of the studies employing multiple time points, one initially found a significant relationship at the first assessment, which was no longer significant at the follow-up points.^31^ One study found no significant relationship at any of the four time points,^35^ while one study found a significant relationship that was maintained across all five time points.^33^

Overall, the most frequently used assessment point was 6 weeks after the dosing session, which was employed in 3 out of the 13 studies.^7,29,34^

## Discussion

This scoping review explored the relationship between mystical-type experiences and treatment outcomes, examining life-threatening disease populations with anxiety and/or depression, compared to depressive clinical populations with anxiety and depression symptoms. We focused on mystical-type experiences because prior research identifies them as a potential mediator of therapeutic outcomes, and because they capture features of the psychedelic state that are distinct from general intensity. Across the 13 clinical trials reviewed (LTD populations, *n* = 5; populations with depression, *n* = 8), we found no substantial difference in the frequency of relationships between mystical-type experiences and treatment outcomes for anxiety and/or depression between these populations. In both groups, mystical-type experiences were generally associated with improvements in anxiety and depression symptoms, with most studies reporting a significant positive relationship between mystical-type experiences and clinical outcomes. In the few studies that did not find such a relationship,^27,35^ participants still demonstrated sustained reductions in depression and/or anxiety symptoms, but these improvements were not statistically associated with mystical-type experience scores. In both LTD and depressive populations, psilocybin was the most commonly administered psychedelic. There were minor differences observed between the two populations in terms of therapeutic support—while all of the studies in LTD populations provided psychotherapy, two of the studies in depressive populations did not.^28,32^ The influence of psychotherapeutic support on the relationship between mystical-type experiences and outcomes was, however, not evident. One of the studies that lacked therapy still found a significant positive relationship,^28^ and the other found a negative relationship.^32^

Our findings are consistent with previous systematic reviews and meta-analyses that often report positive associations between measures of mystical-type experiences and clinical outcomes.^21,37^ However, our review also highlights that this relationship is not universal, as some studies in the present review found no significant association between mystical-type experiences and symptom improvement. This is consistent with findings from Kangaslampi’s systematic review,^20^ which suggests that other aspects of the psychedelic experience, such as emotional breakthroughs and psychological insights, may be equally or more strongly associated with treatment outcomes. Although other aspects of the psychedelic experience may be more influential in predicting treatment outcomes, factors such as the timing of clinical symptom assessment and the therapeutic context of the experience may also contribute to the findings.

The timing of when the relationship between mystical-type experiences and mood outcomes is measured might be relevant to whether a significant relationship is found. Across the studies included in this review, a variety of time points were employed to assess clinical symptoms, from 1 day after dosing to 4.5 years later. Two studies^27,31^ initially found a significant relationship between mystical-type experiences and treatment outcomes, which was no longer significant when assessed at the long-term follow-up points. In one study, the significant relationship found at 4-weeks after dosing was absent 3-, 6-, and 12-months later.^31^ In the other study, there was a significant relationship 6-weeks post dosing,^7^ which was no longer significant at the 4.5 year follow-up.^27^ This suggests that, while mystical-type experiences might be predictive of initial treatment response, they could be less important in determining whether these responses persist over time. It is also possible that the association between mystical-type experiences and treatment outcomes was no longer significant at a later time point because of the benefits of psychedelic-assisted therapy diminishing over time. However, one study found that the correlation between mystical-type experiences and mood symptoms persisted up until the last follow-up point, 6-months post dosing.^33^ These mixed results make it difficult to draw conclusions about whether a significant relationship mediates long-term reductions in depression or anxiety symptoms. While many studies conduct long-term follow-ups, the correlation between the acute psychedelic experience and persisting treatment effects is not often explored. Future studies should aim to include these analyses to determine the significance of different aspects of the acute experience in mediating long-lasting improvements in clinical symptomatology.

The majority of studies in this review were conducted in similar dosing environments, typically utilizing comfortable rooms with curated aesthetics, pre-selected playlists, and the use of eyeshades and headphones. Although this consistency in setting is helpful in standardizing conditions across trials, it limits our ability to determine how context may influence the occurrence of mystical-type experiences and their relationship to clinical outcomes. One study^28^ stood out for administering psychedelics in a notably different environment—specifically, an MRI scanner. Although the study still reported a relationship between mystical-type experiences and clinical outcomes, it involved ketamine, a dissociative rather than a classical psychedelic, which may affect the extent to which environmental context shapes the experience. Notably, the two studies that did not find a relationship between psilocybin-induced mystical-type experiences and clinical outcomes used the same therapeutic context as those that did,^30,35^ making it difficult to definitively determine the role of context. Of these two studies, one^30^ found a correlation between mystical-type experiences and reduction in depression following placebo, but not psilocybin, suggesting that certain therapeutic contexts may increase the likelihood of reporting mystical-type experiences, regardless of pharmacological effects. Considering this, future studies should systematically explore how the environment or “setting” in psychedelic-assisted therapy trials may influence the quality of the psychedelic experience, as well as clinical outcomes. In addition to the physical environment, the beliefs, language, or therapeutic approach of therapists and monitors can significantly influence participants’ “mindset” and may be equally as important in shaping the psychedelic experience and mediating clinical outcomes. Therefore, gaining a better understanding of how various factors of the therapeutic context may influence clinical outcomes is essential for understanding the mechanisms through which psychedelic-assisted therapies produce their therapeutic effects.

## Limitations

This review poses a few limitations. First, although the aim was to compare two populations, namely participants with a life-threatening disease and participants with anxiety and depression symptoms not associated with an LTD, there was an unequal number of studies representing the two populations. Thus, differences between LTD populations and other populations might have been missed due to the smaller sample size of included LTD studies. Second, statistical comparisons between the two populations were not conducted because of the nature of the scoping review process. Future studies could employ a meta-analysis approach to reveal any significant statistical differences between different study populations.

## Conclusion

This review compared patients with a life-threatening disease diagnosis to depressive clinical populations and assessed the relationship between mystical-type experiences induced by psychedelics and depression and anxiety outcomes. No obvious differences in the frequency of observed relationships between mystical-type experiences and treatment outcomes emerged between these two populations, with most studies finding a positive relationship. Although it is possible that mystical-type experiences present different features in patients facing a terminal diagnosis, the available data do not capture these specific details. While mystical-type experiences appear to be commonly associated with improvements in anxiety and depression in various clinical populations, this relationship is likely influenced by multiple factors including the type of psychedelic, the timing of outcome assessment, and the therapeutic context.

## Supplemental Material

sj-docx-1-tpp-10.1177_20451253261433836 – Supplemental material for A scoping review of mystical-type experiences and mood symptom outcomes in psychedelic therapy clinical trials: comparing life-threatening disease and depressive populations Supplemental material, sj-docx-1-tpp-10.1177_20451253261433836 for A scoping review of mystical-type experiences and mood symptom outcomes in psychedelic therapy clinical trials: comparing life-threatening disease and depressive populations by Ana Deutsch, Luis E. Contreras, Sarah Kratina and Leah M. Mayo in Therapeutic Advances in Psychopharmacology

## Declarations

**Ethics approval and consent to participate:** Not applicable (no original data were collected).

**Consent for publication:** Not applicable (no original data were collected).

**Availability of data and materials:** No original data collected.
